# An investigation of psychoactive polypharmacy and related gender-differences in older adults with dementia: a retrospective cohort study

**DOI:** 10.1186/s12877-023-04353-8

**Published:** 2023-10-20

**Authors:** Shanna C Trenaman, Jack Quach, Susan K Bowles, Susan Kirkland, Melissa K Andrew

**Affiliations:** 1 College of Pharmacy, Faculty of Health, Dalhousie University, 5968 College Street, PO Box 15000, B3H 4R2 Halifax, NS Canada; 2 Division of Geriatric Medicine, Department of Medicine, Dalhousie University, Halifax, Canada; 3 Faculty of Health, Dalhousie University, Halifax, NS Canada; 4 Nova Scotia Health, Halifax, NS Canada; 5 Department of Community Health and Epidemiology, Dalhousie University, Halifax, NS Canada

**Keywords:** Dementia, Polypharmacy, Geriatrics, CNS-active medication, Psychoactive medication

## Abstract

**Background:**

Older adults living with dementia may express challenging responsive behaviours. One management strategy is pharmacologic treatment though these options often have limited benefit, which may lead to multiple treatments being prescribed.

**Methods:**

The aim of the present study was to describe psychoactive medication polypharmacy and explore factors associated with psychoactive polypharmacy in a cohort of older adults living with dementia in Nova Scotia, Canada, including a gender-stratified analysis. This was a retrospective cohort study of those aged 65 years or older with a recorded diagnosis of dementia between 2005 and 2015. Medication dispensation data was collected from April 1, 2010, or dementia diagnosis (cohort entry) to either death or March 31, 2015 (cohort exit). Psychoactive medication claims were captured. Psychoactive medication polypharmacy was defined as presence of three or more psychoactive prescription medications dispensed to one subject and overlapping for more than 30 days. Psychoactive polypharmacy episodes were described in duration, quantity, and implicated medications. Regression analysis examined factors associated with experience and frequency of psychoactive polypharmacy. All analysis were stratified by gender.

**Results:**

The cohort included 15,819 adults living with dementia (mean age 80.7 years; 70.0% female), with 99.4% (n = 15,728) receiving at least one psychoactive medication over the period of follow-up. Psychoactive polypharmacy was present in 19.3% of the cohort. The gender specific logistic regressions demonstrated that for both men and women a younger age was associated with an increased risk of psychoactive polypharmacy (women: OR 0.97, 95%CI[0.96, 0.98], men: OR 0.96, 95%CI[0.95, 0.97]). Men were less likely to experience psychoactive polypharmacy if their location of residence was urban (OR 0.86, 95%CI[0.74, 0.99]). There was no significant association between location of residence (urban or rural) and psychoactive polypharmacy for women living with dementia. Antidepressants were the most dispensed medication class, while quetiapine was the most dispensed medication.

**Conclusions:**

This study suggests that of adults living with dementia those of younger ages were more likely to experience psychoactive polypharmacy and that men living with dementia in rural locations may benefit from increased access to non-pharmacological options for dementia management.

**Supplementary Information:**

The online version contains supplementary material available at 10.1186/s12877-023-04353-8.

## Background

Dementia is a growing concern for global healthcare systems with effects extending to people living with dementia (PLWD), their carers, and the health and social care systems supporting them [1]. Dementia is the second leading cause of death in those aged over 70 years and was implicated in the loss of 28.8 million disability-adjusted life-years in 2016 [1]. In 2020 estimates were that 597,300 adults lived with dementia in Canada, a number which is expected to increase to over 1 million by 2030. In Nova Scotia, an eastern province of Canada, estimates suggest that at any point in time 1.6% of the population (approximately 17,000 individuals) are PLWD, projected to grow 80% greater by the year 2050 [2]..

Among the many challenges associated with the management of dementia is the prevalence of responsive behaviours; estimated to occur in 25% of PLWD [3–5]. Responsive behaviours are defined as verbal or physical actions that can be disruptive, distressing or challenging to persons in the environment [6]. There is substantial variation in the presentation of responsive behaviours [7, 8] and medications are often prescribed for their management [9–14]. Investigation into the effect of pharmacotherapy to manage responsive behaviours in PLWD has demonstrated limited benefit [15, 16]. Continued reliance on ineffective medication treatments for responsive behaviours may contribute to polypharmacy as multiple psychoactive medications are trialed. Use of psychoactive medications has been linked to increased risk of falls [17–19], a paradoxical increase in responsive behaviours, worsening cognition, stroke [20], and death [21]. Age, sex [22, 23], and rurality [24] are related to dementia risk and may play a role in the likelihood of psychoactive medications being prescribed and the potential for psychoactive medication polypharmacy in PLWD. Prescribing guidelines advocate for limiting exposure to psychoactive medications in managing responsive behaviors in PLWD [25–27]. In order to reduce inappropriate use of psychoactive agents in PLWD, it is important to understand which factors are associated with their use. This may allow for targeted interventions for those at the greatest risk.

The objectives of this study were to (1) describe psychoactive polypharmacy, defined as use of three psychoactive medications for 30 days or more, in a cohort of older adults living with dementia in Nova Scotia, Canada and (2) investigate the association of gender, age, and rurality with psychoactive polypharmacy and the number of psychoactive polypharmacy episodes, including a gender-stratified analysis.

## Methods

### Cohort identification

The study population was drawn from the Seniors’ Pharmacare beneficiaries in the province of Nova Scotia (NS), Canada. The Seniors’ Pharmacare program is a voluntary government funded prescription medication plan for adults 65 years of age and older in NS. Seniors’ Pharmacare is available to and used by both long-term care (LTC) dwelling and community-dwelling adults. Seniors’ Pharmacare beneficiaries with a diagnosis of dementia were selected and while it was not possible to tell on an individual level whether they were community-dwelling or residents of LTC, both were included in the dataset and analyses. The definition of dementia was taken from the recommendations of the NS Dementia Strategy to identify dementia in administrative health data in the province [28]. Dementia was identified when a subject had at least one entry in the health administrative data of a code associated with dementia. Relevant dementia-related codes are listed in Table 1. The administrative datasets consulted included the Medical Services Insurance database which catalogues ambulatory care visits including details of visits with primary care providers and the Canadian Institute for Health Information Discharge Abstract database which catalogues emergency department and hospital visits. Cohort entry was assessed from 1 to 2005 to 30 March 2015 for any occurrence of the relevant ICD9/10 codes (Table 1). At identification of a dementia related code the Seniors’ Pharmacare beneficiary was entered into the cohort (See Appendix 1). The cohort subjects were followed until censoring by death or the end of the period of observation (30 March 2015).

**Table 1:** Dementia ICD9/10 diagnosis codes used as case definition for cohort identification

Description	ICD-9	ICD-10
Alcohol-induced persisting amnestic disorder	290.x	F01.x, F05.1
Alcohol-induced persisting dementia	291.1	F10.6
Amnestic disorder in conditions classified elsewhere	291.2	F10.7
Dementia in conditions classified elsewhere	294.0	F04.x
Other cerebral degenerations ***Includes***: *Alzheimer’s disease; Frontotemporal dementia; Senile degeneration of the brain; Communicating hydrocephalus; Idiopathic normal pressure hydrocephalus; Cerebral degeneration in diseases classified elsewhere; dementia with Lewy’s bodies; Dementia with Parkinsonism; Cerebral degeneration, unspecified.* ***Excludes***: *Obstructive hydrocephalus; Reye’s syndrome*	331.0-331.3, 331.5-331.7, 331.82, 331.83, 331.89, 331.9	G30.x, G31.0, G31.1, G31.8, G31.9, G32.8, G91.0, G91.2-G91.3, G91.8, G91.9, G94.x
Senility without mention of psychosis	797	R54.x

from [28]

### Psychoactive medication polypharmacy identification

Use of medications from the following four classes; antidepressants (tricyclic antidepressants, selective serotonin reuptake inhibitors (SSRIs), bupropion and trazodone), cholinesterase inhibitors, antipsychotics (first- and second-generation), benzodiazepines, and nonbenzodiazepine benzodiazepine receptor agonist hypnotics (z-drugs)) was reported. Psychoactive medications from these classes were chosen as those more commonly used to manage behavioural symptoms of dementia and this data were available for analysis from another cohort study [29–31]. Data were not available for some other medications that could have been relevant to the analysis, including the atypical antidepressant mirtazapine, the selective norepinephrine reuptake inhibitor duloxetine, and anticonvulsants. Medication use was approximated from the dispensing data recorded in the Seniors’ Pharmacare database (PHARM). Exposure to a medication was defined as any dispensation according to the PHARM record, with the required assumption that dispensation was equivalent to medication use. Medication related data was tracked over the five fiscal years from 1 April 2010 to 30 March 2015. Medication use data included ATC code, prescription fill date, and days supplied, along with sociodemographic characteristics of the beneficiary including age at dementia diagnosis, date of dementia diagnosis, gender, urban or rural location of residence [32], and date of death if it occurred during the period of observation. Urban and rural location of residence were determined by the second digit of the postal code which denotes an urban location (city or town) when expressed as a non-zero number and a rural location when the second digit of the postal code was zero. Daily medication exposure was estimated from the prescription fill date and the days supplied of the prescription.

### Statistical analysis

Prescription data was reported at the level of the ATC code/generic name. We reported mean (standard deviation; SD) age at dementia diagnosis. Using dispensation date and days supplied we identified duration of each prescription medication from the specified list of psychoactive medications for assessment. We then identified medication use that overlapped by assessment of the dates the medications were dispensed and therefore available to the subject. Presence of three or more concomitant psychoactive medications dispensed and available on a specific day was noted and when this continued for more than 30 days it was captured as an episode of psychoactive polypharmacy.

Given known sex and gender differences in medication use in dementia [16], we reported psychoactive polypharmacy for the cohort and for men and women in a gender-stratified analysis which included independent t-tests to compare age of the men and women in the cohort and to compare duration of psychoactive polypharmacy. As age at diagnosis may be related to the duration of dementia we expanded the regression model to include year of diagnosis as a variable. We reported common drugs and drug pairings in psychoactive polypharmacy. We used logistic regression to assess for risk factors for psychoactive polypharmacy. Covariables for assessment included age at dementia diagnosis, gender, year of dementia diagnosis, and location of residence (urban or rural). Ability of the logistic regression model to predict the outcome was assessed by calculating the area under the curve. Negative binomial hurdle regression models were used to investigate the association between age at dementia diagnosis, sex, location of residence (urban or rural), and year or dementia diagnosis with the total number of psychoactive polypharmacy events. In brief, negative binomial hurdle regression models have two components, one specifying for zero counts (similar to logistic regression) and another specifying for positive counts. This type of model accounts for the zero-inflated nature (expectation of many zero-valued observations) of the psychoactive polypharmacy definition. A plot of residuals was completed to assess the validity of the hurdle regression models. For both the logistic regression and hurdle regression we had three models for each gender assessed starting with (1) age, (2) age and rurality and (3) age, rurality, and year of dementia diagnosis for a total of 12 models.

Data processing and analyses were performed using R version 4.0.5. The Nova Scotia Health Research Ethics Board approved this study (REB File #: 1,023,625). Informed consent was waived due to the use of de-identified administrative data.

## Results

### Prevalence of psychoactive polypharmacy

**Fig. 1:**
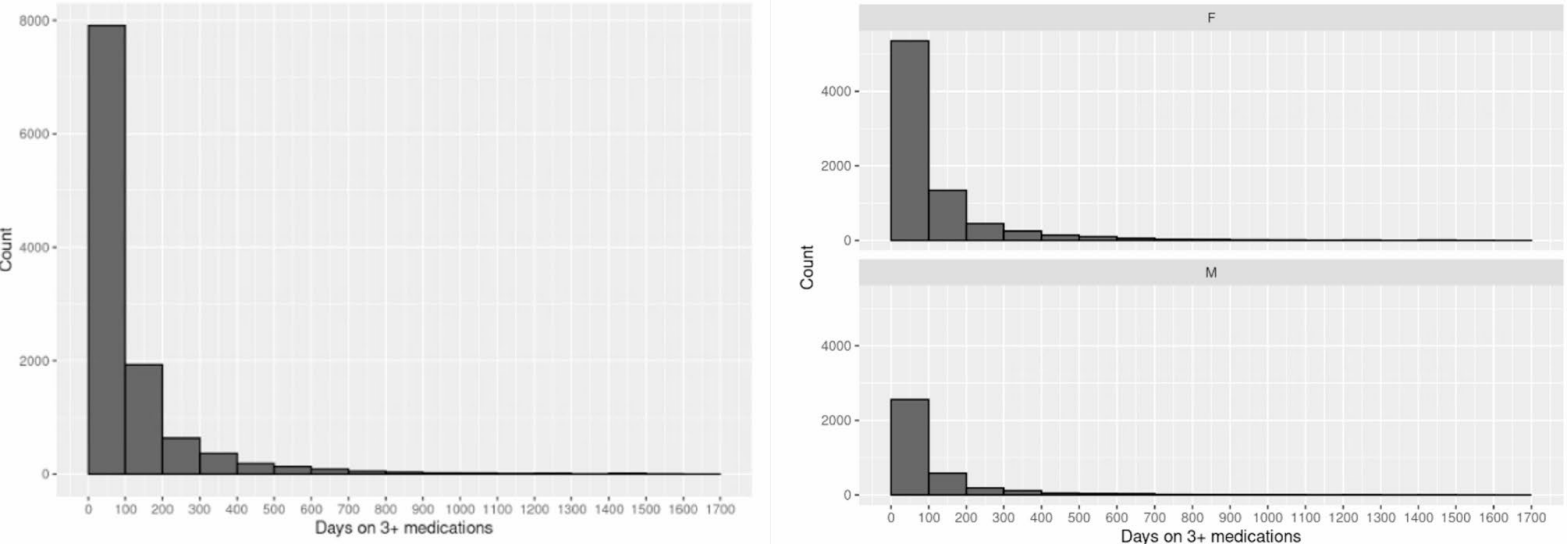
Number of psychoactive polypharmacy events overall with comparison of men and women for older adults living with dementia

The cohort included 15,819 older adults with dementia. The cohort was comprised of subjects with a recorded diagnosis of dementia between 2005 and 2015. The mean age at dementia diagnosis was 80.7 (SD 7.8 years) and 70.0% were female. The mean age at dementia diagnosis was older for women compared to men at 81.9 years (SD 7.8 years) for women and 78.0 years (SD 7.3 years) for men (p < 0.001). We identified that 99.4% (n = 15,728) of the cohort were receiving at least one psychoactive medication over the period of follow-up and 3,054 unique subjects had at least one episode of psychoactive medication polypharmacy (19.3%). Figure 1 shows the comparison of men and women with respect to psychoactive polypharmacy events. The results demonstrated that women had more psychoactive polypharmacy events than men (n = 2,090 (women) versus n = 964 (men)), however, the proportion of men having a psychoactive polypharmacy event was similar to women (20.2% for men and 19.0% for women (p = 0.06)) due to the preponderance of women in the cohort.

### Duration of psychoactive polypharmacy

**Fig. 2:**
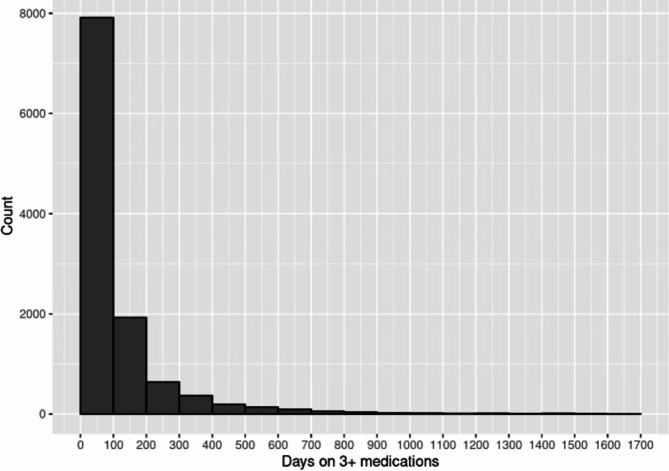
Duration (days) of psychoactive polypharmacy events in a cohort of older adult living with dementia in Nova Scotia, Canada

The mean duration of psychoactive polypharmacy events was 111 (SD 245) days for women and 99.3 (SD 224) days for men (p = 0.004). Figure 2 shows the duration in days of psychoactive polypharmacy events. The  38.6% of events lasted less than 100 days, though notably 1,866 psychoactive polypharmacy events (61.1% of all events) exceeded 100 days and 384 (12.6%) psychoactive polypharmacy events exceeded 500 days. Table 2 shows the characteristics of the cohort of PLWD based on duration of psychoactive polypharmacy.

**Table 2:** Description of the cohort of people living with dementia and those who experience psychoactive polypharmacy by duration of psychoactive polypharmacy

	Entire Cohort	No psychoactive polypharmacy	Psychoactive polypharmacy < 100 days	Psychoactive polypharmacy 100–500 days	Psychoactive polypharmacy > 500 days
N	15,819	12,765	1,179	1,491	384
Women (%)	11,051 (69.9)	8,961 (70.2)	772 (65.5)	1044 (70.0)	274 (71.3)
Men (%)	4,768 (30.1)	3,804 (29.8)	407 (34.5)	447 (30.0)	110 (28.6)
Urban (%)	9,968 (63.0)	8,075 (63.3)	759 (64.3)	901 (60.4)	233 (60.7)
Age, in years (SD)	80.7 (7.83)	81.1 (7.85)	79.2 (7.5)	79.1 (7.5)	78.5 (7.71)

### Most frequently dispensed medications in those with psychoactive polypharmacy

The top 10 medications that were present in psychoactive polypharmacy were citalopram (19.3% of psychoactive polypharmacy episodes), trazodone (15.5%) quetiapine (11.4%), donepezil (9.4%), risperidone (8.0%), galantamine (5.9%), zopiclone (5.8%), venlafaxine (4.7%), sertraline (3.5%), and paroxetine (3.1%). The 10 most common psychoactive drug pairings were: citalopram and trazodone, quetiapine and trazodone, risperidone and trazodone, quetiapine and citalopram, and risperidone and citalopram, citalopram and donepezil, trazodone and donepezil, zopiclone and citalopram, zopiclone and trazodone, and quetiapine and risperidone. Table 3 shows the frequency and proportion of these most common pairings that were identified in those with psychoactive polypharmacy.

**Table 3:** Most common drug-drug combinations prescribed to people living with dementia and experiencing a period of psychoactive polypharmacy

Drug Combinations	Frequency	Proportion
citalopram & trazodone	2,251	0.060
quetiapine & trazodone	1,703	0.046
risperidone & trazodone	1,554	0.042
quetiapine & citalopram	1,464	0.039
risperidone & citalopram	1,243	0.033
citalopram & donepezil	1,210	0.033
trazodone & donepezil	1,009	0.027
zopiclone & citalopram	980	0.026
zopiclone & trazodone	962	0.026
quetiapine & risperidone	882	0.024

### Risk factors for psychoactive polypharmacy

Logistic regression adjusted for age at dementia diagnosis, year of dementia diagnosis, and rural or urban location of residence demonstrated for the cohort overall and both for men and women in the gender-stratified analyses that younger age was associated with increased risk of psychoactive polypharmacy (overall: 0.97, 95%CI[0.96, 0.97], women: OR 0.97, 95%CI[0.96, 0.98], men: OR 0.96, 95%CI[0.95, 0.97]) (See Table 4). Men were more likely to experience psychoactive polypharmacy if their location of residence was rural (OR 0.86, 95%CI[0.74, 0.99]), but there was no significant association between location of residence (urban or rural) and polypharmacy for women living with dementia. Diagnosis of dementia in 2010, 2011, 2012, 2013, 2014 and 2015 were associated with a reduced risk of psychoactive polypharmacy compared to diagnosis in 2005 (Table 4) with some variation for men and women. Area under the curve for the regression specifically for men suggested fair model predictive ability (0.631), and for women slightly less so (0.618). We did not find an interaction between gender and age. Hurdle models (Table 4) showed that being one year older at diagnosis was significantly associated with 2% lower total number of psychoactive polypharmacy events (OR 0.98, 95%CI[0.97, 0.99]). Visual inspection of the plot of residuals for the hurdle models suggested no concerns of heteroscedasticity, outliers, or unequal distribution around the zero line, but there was some potential for a slight negative drift in the residual plot suggesting the model may not capture all key factors and there may be an underprediction of the count. Additional regression findings are shown in Table 4.

**Table 4:** Logistic regression and hurdle regression results for risk factors for psychoactive polypharmacy in older men and women with dementia

Variable	Overall OR (95% CI)	Men OR (95% CI)	Women OR (95% CI)
***Logistic Regression***			
Age at diagnosis	**0.97 (0.96, 0.97)** ^*^	**0.96 (0.95, 0.97)** ^*^	**0.97 (0.96, 0.98)** ^*^
Year of diagnosis 2005	Referent		
Year of diagnosis 2006	1.08 (0.90, 1.30)	1.29 (0.90, 1.83)	1.00 (0.80, 1.25)
Year of diagnosis 2007	1.18 (0.99, 1.30)	0.87 (0.61, 1.25)	1.31 (1.06, 1.61)
Year of diagnosis 2008	0.96 (0.81, 1.14)	0.89 (0.64, 1.24)	1.16 (0.95, 1.42)
Year of diagnosis 2009	0.87 (0.74, 1.03)	0.84 (0.61, 1.16)	1.01 (0.83, 1.23)
Year of diagnosis 2010	**0.71 (0.60, 0.84)** ^*^	0.80 (0.59, 1.09)	0.90 (0.74, 1.09)
Year of diagnosis 2011	**0.71 (0.60, 0.84)** ^*^	**0.69 (0.50, 0.95)** ^*^	**0.71 (0.57, 0.87)** ^*^
Year of diagnosis 2012	**0.62 (0.52, 0.74)** ^*^	**0.41 (0.29, 0.58)** ^*^	**0.74 (0.60, 0.91)** ^*^
Year of diagnosis 2013	**0.55 (0.46, 0.66)** ^*^	**0.50 (0.35, 0.70)** ^*^	**0.57 (0.46, 0.72)** ^*^
Year of diagnosis 2014	**0.39 (0.32, 0.48)** ^*^	**0.45 (0.32, 0.64)** ^*^	**0.34 (0.26, 0.45)** ^*^
Year of diagnosis 2015	**0.29 (0.19, 0.44)** ^*^	**0.17 (0.06, 0.36)** ^*^	**0.37 (0.22, 0.58)** ^*^
Urban	0.95 (0.87, 1.03)	**0.86 (0.74, 0.99)** ^*^	0.99 (0.89, 1.09)
***Hurdle Regression***			
Age at diagnosis	**0.98 (0.98, 0.99)** ^*^	**0.98(0.97, 0.99)** ^*^	**0.98 (0.97, 0.99)** ^*^
Year of diagnosis 2005	Referent		
Year of diagnosis 2006	0.98 (0.77, 1.23)	1.15 (0.76, 1.76)	0.94 (0.71, 1.24)
Year of diagnosis 2007	0.95 (0.76, 1.20)	1.33 (0.87, 2.08)	0.86 (0.66, 1.12)
Year of diagnosis 2008	0.99 (0.79, 1.24)	1.04 (0.69, 1.57)	0.99 (0.76, 1.29)
Year of diagnosis 2009	0.97 (0.78, 1.20)	1.15 (0.77, 1.71)	0.92 (0.71, 1.19)
Year of diagnosis 2010	0.91 (0.74, 1.12)	1.25 (0.86, 1.83)	0.80 (0.62, 1.04)
Year of diagnosis 2011	0.88 (0.70, 1.10)	1.13 (0.76, 1.68)	0.81 (0.62, 1.07)
Year of diagnosis 2012	**0.70 (0.55, 0.89)** ^*^	0.95 (0.60, 1.48)	**0.64 (0.48, 0.85)** ^*^
Year of diagnosis 2013	**0.61 (0.48, 0.79)** ^*^	0.72 (0.47, 1.12)	**0.59 (0.43, 0.80)** ^*^
Year of diagnosis 2014	**0.55 (0.41, 0.73)** ^*^	0.83 (0.52, 1.32)	**0.43 (0.30, 0.64)** ^*^
Year of diagnosis 2015	0.57 (0.31, 1.04)	0.67 (0.20, 2.30)	0.54 (0.27, 1.07)
Urban	1.10 (0.98, 1.22)	**1.22 (1.01, 1.48)** ^*^	1.05 (0.91, 1.20)

* Signifies a statistically significant result

## Discussion

In this large cohort of PLWD from a provincially funded prescription drug program, psychoactive medication polypharmacy defined as exposure to three psychoactive medications for 30 days or more was present in 19.3% of subjects. Among those who experienced psychoactive polypharmacy, citalopram was the most dispensed psychoactive medication, being dispensed to 19.3% of subjects. The finding that an antidepressant was the most dispensed class of medication had both positive and negative considerations. A positive interpretation is that selective serotonin reuptake inhibitors (SSRIs) are likely to have fewer central nervous system related adverse effects than other antidepressants or antipsychotics [33]. Additionally, most SSRIs are less anticholinergic than tricyclic antidepressants (which is favourable for older adults with dementia) and may be better tolerated with less risk of precipitating further cognitive impairment. However, SSRIs are not without risks and are known, for example, to be associated with risks of falls and fractures, and citalopram is also associated with QT prolongation [34–36]. Trazodone was second in frequent dispensation to those with an episode pf psychoactive polypharmacy and is frequently used for sleep or agitation despite a lack of established research confirming safety and effectiveness [37]. While we were not able to capture dose or indication it is likely that some significant portion of trazodone dispensation was intended to support sleep or agitation (especially in the evening). Like other antidepressants there are risks associated with its use [10, 11, 33] for older adults with dementia. Notably, the combination of trazodone and zopiclone was ninth on the top 10 list of combinations in our study; this may represent concerning use of two medications for one indication (sedation).

Quetiapine was implicated in 11.4% of episodes of psychoactive polypharmacy. As the third most dispensed medication to those experiencing psychoactive polypharmacy its use contrasts with prescribing recommendations which advise against antipsychotic use in older adults with dementia. These guidelines have not been able counter the perception of relative safety of quetiapine amongst prescribers which may contribute to its high level of use [38, 39]. Prescribers may perceive that low-dose quetiapine is safe and effective in treating responsive behaviours, despite mounting evidence of potential harm and limited evidence of benefit [38, 39]. Of some concern the tenth most common pair of drugs implicated in psychoactive polypharmacy included both quetiapine and risperidone – two antipsychotics – which is likely to cause more antipsychotic related harms than benefits. Antipsychotic polypharmacy does not have proven benefit in treating PLWD. It is possible that the overlapping of these two antipsychotics reflects drug switching although it is not possible for us to determine this with the data collected.

Overall, benzodiazepines were dispensed at least once to 28.2% of the cohort over the five-year period of study. Zopiclone was dispensed to 10.3% of the cohort over the study period (Appendix 2). While benzodiazepines were not in the top medications implicated in psychoactive polypharmacy, the nonbenzodiazepine benzodiazepine receptor agonist hypnotic zopiclone was used by 5.8% of the cohort experiencing psychoactive polypharmacy. In 2018 Nova Scotia placed third in Canada with respect to the most benzodiazepine dispensations [40]. Use of benzodiazepines has been reported to be common in other studies as well, for example they were used by 9.3% of community-dwelling older adults in the United States Medication Expenditure Panel Study [25]. Benzodiazepine use was common in Nova Scotia, and was common for PLWD in our cohort. Relatively low levels of benzodiazepine dispensation as part of psychoactive polypharmacy in our cohort of older adults with dementia likely reflects messaging about the risks of benzodiazepines in older adults and may lead to more cautious medication use when benzodiazepines are used.

Rurality was associated with psychoactive polypharmacy for older men living with dementia but not women. This suggests that prescribers in rural locations may need support managing responsive behaviours for men living with dementia in rural locations and may need increased access to non-pharmacological options for dementia management. Previous reports from Quebec suggest that there are differences for those living with dementia when comparing those residing in an urban or rural location - rural residents living with dementia had higher mortality, less physicians access, more hospitalizations, shorter hospital stays, higher antipsychotic medication use, lower use of home care services, and higher use of nursing home compared to urban persons living with dementia [41]..

Those PLWD with a later (more recent) diagnosis of dementia were found to have a reduced risk of psychoactive polypharmacy. Those diagnosed later were more likely to have fewer years living with the illness, had less time for cognition to deteriorate, and therefore likely had fewer responsive behaviours that needed treatment. It is also a consideration that messaging and continuing professional education about limited benefit and increased risks associated with using pharmacotherapy to treat responsive behaviours is being accepted by prescribers. Successful knowledge translation may be allowing those diagnosed with dementia more recently to avoid prescription of psychoactive medications. Conversely those diagnosed in earlier years may have already been established on psychoactive medications and it may have been challenging to know whether the treatments should be discontinued or not and they ended up being continued. Further research into deprescribing psychoactive medication in PLWD would provide the confidence in prescribers and represents an avenue of needed study.

Drug combinations that were implicated as components of psychoactive polypharmacy were of interest. Citalopram and trazodone, the most common pair, are both serotonergic agents and could lead to an increased risk serotonin syndrome or QT interval prolongation; at higher doses. Monitoring details for either of these complications and risks of these drug combinations are likely complicated for PLWD and their carers. Additionally, quetiapine and trazodone, risperidone and trazodone, quetiapine and citalopram, and risperidone and citalopram all are potential QT interval prolonging drug-drug interactions. Targeted information materials about QT interval prolongation may be needed for this population. Identifying two atypical antipsychotics as the tenth most common drug pairing in psychoactive polypharmacy is of concern given unclear benefit and considerable risk associated with antipsychotics use by PLWD. Falls, stroke, and mortality are all risks associated with antipsychotics [33] and there is no evidence that combination of antipsychotics provide benefit for PLWD.

The questions that remain are whether and why the use of multiple psychoactive medications was needed. We cannot report on indication, but the hypotheses considered were that either solitary medications were not successful for management of targeted symptoms or there were multiple symptoms or behaviours to target. With more psychoactive medication polypharmacy among the younger people in the cohort it suggests that either the younger members of the cohort have more significant symptoms, can request treatment being earlier into disease progression, or are seen as more challenging to handle with their more robust bodies. Without high quality evidence to guide medication choices it is unlikely that younger adults with dementia will benefit from pharmacotherapy and in fact they may then experience more medication-related adverse events or risks due to their increased exposure. Simply expecting that younger adults with dementia will tolerate more medication adverse effects is not acceptable; supported management strategies that focus firstly on non-pharmacologic interventions are preferred and should be readily available to those living with dementia.

We expect that some presently unknown level of psychoactive polypharmacy is appropriate for a cohort of PLWD. However, it remains unclear what that level is. We should assume that the shortest duration of exposure to psychoactive polypharmacy is beneficial to prevent complications from multiple drug exposure. Frequent medication review for those receiving multiple psychoactive medications concurrently is a reasonable approach. We suspect that this was the case as there were many psychoactive polypharmacy events in fewer subjects suggesting that trials of more psychoactive medications were prescribed and deprescribed over the course of clinical care. Recognizing 90 days as an ideal time-point for medication review when multiple psychoactive medications are prescribed to a PLWD in a dynamic disease which will have changing needs is an appropriate benchmark in practice. This may be best shared as a responsibility of all healthcare providers in a patient’s circle of care including nursing, pharmacy, and physician or nurse practitioner prescribers.

### Limitations

There was no access to clinical data which would have allowed assessment of the appropriateness of the many therapies reported. It was not possible to assess actual ingestion and adherence, nor whether medications were taken as prescribed. Drug dosage was not collected and therefore in addition to an inability to comment on the appropriateness of a particular drug the dose was also unable to be assessed. A previous study has suggested that mirtazapine is one of the most commonly used antidepressants in older adults with dementia [42]. Data for mirtazapine, duloxetine, and antiepileptics were not reported which makes it likely that we are underestimating psychoactive polypharmacy.

Over time the cohort size shrunk due to death or subjects moving away from the province. We also must acknowledge that the cohort gets younger over time due to older people who had been identified in the earliest years of case identification passing away, which may affect the behaviour of the cohort. While we used the definition of dementia that was developed by the NS dementia strategy to identify dementia cases from routinely collected administrative data it is still possible that we missed identification of some individuals due to under-diagnosis which unfortunately remains common with dementia. We also did not have access to in-hospital use of medication where multiple psychoactive medications may have been used together during the hospital stay. We are unable to account for location of residence and recognize that those residing in the community and those residing in a long-term care facility may have differing likelihoods of psychoactive medication use. The slight negative drift in the plot of residuals from the hurdle regression may suggest that there was a missing predictor variable. We do not believe the slight negative drift was significant, we expect it was unlikely to affect the findings, and would likely only cause moderating or underestimating of the counts. Adding additional factors to the regression would increase model complexity, making it harder to interpret and increase the risk of overfitting, without necessarily leading to a significant improvement in predictive performance.

## Conclusions

Psychoactive polypharmacy was present in 19.3% of the cohort of older adults with dementia. This report reviewed psychoactive medications from only four classes; (1) antidepressants (tricyclic antidepressants, SSRIs, bupropion and trazodone), cholinesterase inhibitors, (2) antipsychotics (first- and second-generation), (3) benzodiazepines, and (4) z-drugs which suggests that our estimates of psychoactive polypharmacy were underestimated. Older age was associated with a reduced risk of psychoactive medication polypharmacy. Rurality was associated with psychoactive polypharmacy for older men with dementia but not women. Due to concerns about the potential harms of psychoactive medication use, it is of utmost importance that a collaborative team approach to prescribing and deprescribing be used to minimize psychoactive medication exposure among older adults with dementia.

### Electronic supplementary material

Below is the link to the electronic supplementary material.

Supplementary Material 1

Supplementary Material 2

## Data Availability

The data used in this report were made available by Health Data Nova Scotia of Dalhousie University. Although this research is based on data obtained from the Nova Scotia Department of Health and Wellness, the observations and opinions expressed are those of the authors and do not represent those of either Health Data Nova Scotia or the Department of Health and Wellness. Data can be made available upon reasonable request to the corresponding author after necessary approvals by Health Data Nova Scotia.

## List of Abbreviations

ATC: Anatomical Therapeutic Chemical
ICD: International classification of diseases
NS: Nova Scotia
OR: odds ratio
PHARM: Seniors’ Pharmacare database
PLWD: people living with dementia
SD: standard deviation
SSRI: selective serotonin reuptake inhibitor
z: drugs-nonbenzodiazepine benzodiazepine receptor agonist hypnotics
