# Supplementary material for: An investigation of psychoactive polypharmacy and related gender-differences in older adults with dementia: a retrospective cohort study
Source: BMC Geriatr. 2023 Oct 20;23:683. doi: 10.1186/s12877-023-04353-8 (PMC10590009; doi:10.1186/s12877-023-04353-8)
Supplement: Supplementary file 1 — Supplementary Material 1 [file 12877_2023_4353_MOESM1_ESM.docx]

Appendix 1: Patient Flow

15819 older adults with dementia subscribed to Nova Scotia Seniors Pharmacare diagnosed between 2005 and 2015

3054 (19.3%) met definition of polypharmacy

964 (20.2%) men met definition of polypharmacy

2090 (18.9%) women met definition of polypharmacy

4768 (30.1%) men

11051 (69.9%) women
