# Supplementary material for: An investigation of psychoactive polypharmacy and related gender-differences in older adults with dementia: a retrospective cohort study
Source: BMC Geriatr. 2023 Oct 20;23:683. doi: 10.1186/s12877-023-04353-8 (PMC10590009; doi:10.1186/s12877-023-04353-8)
Supplement: Supplementary file 2 — Supplementary Material 2 [file 12877_2023_4353_MOESM2_ESM.docx]

Appendix 2: CNS-active medication use by older adults with dementia in Nova Scotia, Canada from 1 April 2010 to 30 March 2015 presented in annual cohorts and reported for the entire period of study.

| **Year** | **2011** | **2012** | **2013** | **2014** | **2015** | **2010-2015** |
| --- | --- | --- | --- | --- | --- | --- |
| **N** | 19,127 | 17,597 | 15,890 | 14,070 | 12,211 | 28,952 |
| **Sex** | | | | | | |
| **Female, n (%)** | 13,027 (68.1) | 11,939 (67.8) | 10,716 (67.4) | 9,481 (67.4) | 8,183 (67.0) | 17,946 (62.0) |
| **Male, n (%)** | 5,859 (30.6) | 5,411 (30.7) | 4,925 (31.0) | 4,341 (30.9) | 3,785 (31.0) | 10,528 (36.4) |
| **Age, median (IQR)** | 84.0  (78.2, 89.5) | 83.8  (78.0, 89.2) | 83.3  (77.6, 89.0) | 83.4  (77.4, 88.8) | 83.4  (77.4, 88.8) | 82.0*  (75.0, 87.0)* |
| **Rurality** | | | | | | |
| **Urban, n (%)** | 12,446 (65.1) | 11,420 (64.9) | 10,327 (65.0) | 9,148 (65.0) | 7,934 (64.5) | 19,610 (67.7) |
| **Rural, n (%)** | 6,681 (34.9) | 6,177 (35.1) | 5,563 (35.0) | 4,922 (35.0) | 4,277 (35.0) | 9,342 (32.3) |
| **Antidepressant, n (%)** | 7,385 (38.6) | 7,361 (41.8) | 7,000 (44.1) | 6,491 (46.1) | 6,005 (49.2) | 12,100 (41.8) |
| **Bupropion, n (%)** | 184 (1.0) | 203 (1.2) | 197 (1.2) | 183 (1.3) | 185 (1.5) | 392 (1.4) |
| **SSRI, n (%)** | 4,749 (24.8) | 4,894 (27.8) | 4,722 (29.7) | 4,407 (31.3) | 4,091 (33.5) | 8,031 (27.7) |
| **Tricyclic antidepressants, n (%)** | 1,281 (6.7) | 1,155 (6.6) | 980 (6.2) | 881 (6.2) | 730 (6.0) | 2,136 (7.4) |
| **Trazodone, n (%)** | 2,742 (14.3) | 2,816 (16.0) | 2,785 (17.5) | 2,689 (19.1) | 2,538 (20.7) | 6,010 (20.8) |
| **Antipsychotic, n (%)** | 3,568 (18.7) | 3,631 (20.6) | 3,497 (22.0) | 3,342 (23.8) | 3,273 (26.8) | 7,297 (25.2) |
| **1^st^ generation, n (%)** | 702 (3.7) | 736 (4.2) | 701 (4.4) | 677 (4.8) | 696 (5.7) | 2,156 (7.4) |
| **2^nd^ generation, n (%)** | 3,131 (16.4) | 3,194 (18.2) | 3,098 (19.5) | 2,969 (21.1) | 2,909 (23.8) | 6,335 (21.9) |
| **Benzodiazepine, n (%)** | 4,438 (23.2) | 4,115 (23.4) | 3,719 (23.4) | 3,289 (23.4) | 2,798 (22.9) | 8,171 (28.2) |
| **Z-drug, n (%)** | 1,331 (7.0) | 1,362 (7.7) | 1,347 (8.5) | 1,288 (9.2) | 1,153 (9.4) | 2,981 (10.3) |

*Reported age at dementia diagnosis
